# VIGS approach reveals the modulation of anthocyanin biosynthetic genes by *CaMYB* in chili pepper leaves

**DOI:** 10.3389/fpls.2015.00500

**Published:** 2015-07-07

**Authors:** Zhen Zhang, Da-Wei Li, Jing-Hao Jin, Yan-Xu Yin, Huai-Xia Zhang, Wei-Guo Chai, Zhen-Hui Gong

**Affiliations:** ^1^College of Horticulture, Northwest A&F UniversityYangling, China; ^2^State Key Laboratory for Stress Biology of Arid Region Crop, Northwest A&F UniversityYangling, China; ^3^Institute of Vegetables, Hangzhou Academy of Agricultural SciencesHangzhou, China

**Keywords:** *Capsicum annuum* L., purple leaves, *CaMYB*, virus-induced gene silencing, anthocyanin biosynthesis, *P. capsici* resistance

## Abstract

The purple coloration of pepper leaves arises from the accumulation of anthocyanin. Three regulatory and 12 structural genes have been characterized for their involvement in the anthocyanin biosynthesis. Examination of the abundance of these genes in leaves showed that the majority of them differed between anthocyanin pigmented line Z1 and non-pigmented line A3. Silencing of the R2R3-*MYB* transcription factor *CaMYB* in pepper leaves of Z1 resulted in the loss of anthocyanin accumulation. Moreover, the expression of multiple genes was altered in the silenced leaves. The expression of *MYC* was significantly lower in *CaMYB*-silenced leaves, whereas *WD40* showed the opposite pattern. Most structural genes including *CHS, CHI, F3H, F3*′*5*′*H, DFR, ANS, UFGT, ANP*, and *GST* were repressed in *CaMYB*-silenced foliage with the exception of *PAL, C4H*, and *4CL*. These results indicated that *MYB* plays an important role in the regulation of anthocyanin biosynthetic related genes. Besides *CaMYB* silenced leaves rendered more sporulation of *Phytophthora capsici Leonian* indicating that *CaMYB* might be involved in the defense response to pathogens.

## Introduction

**FIGURE 1:**
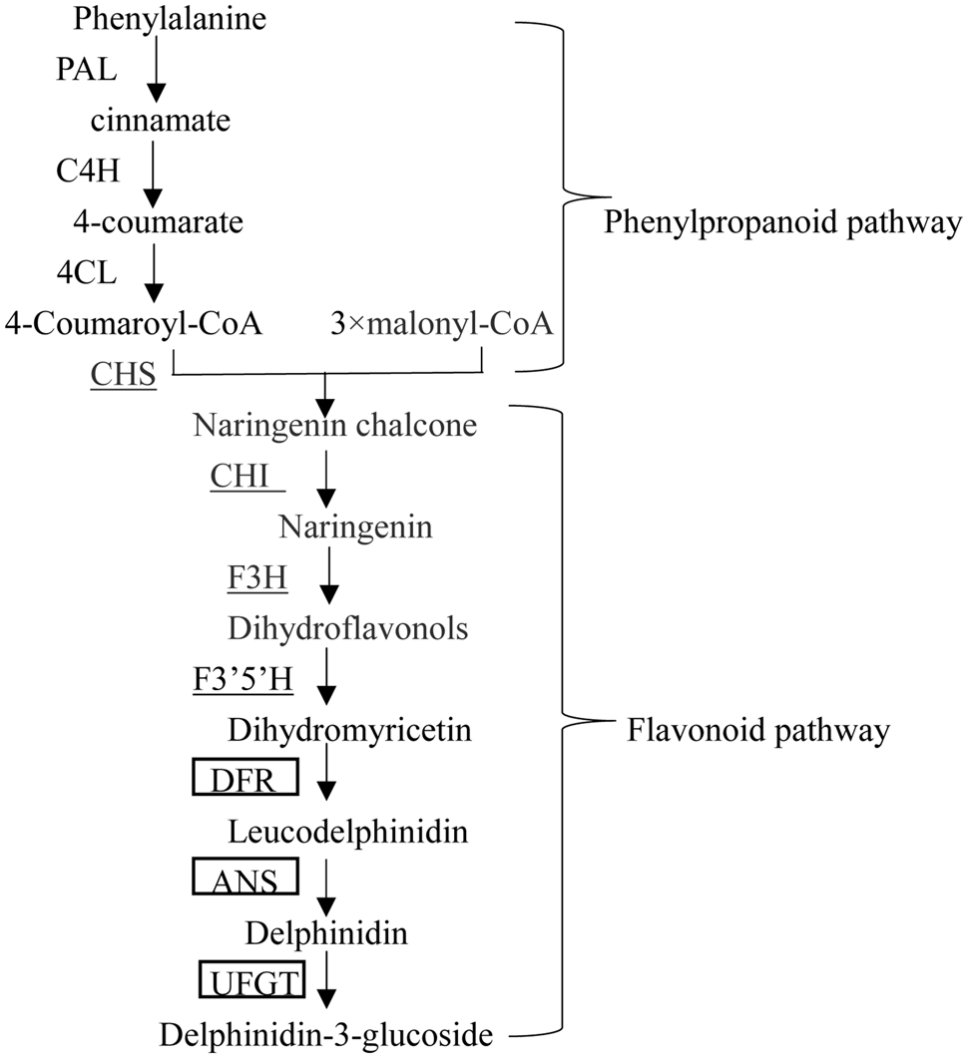
**Anthocyanin biosynthesis pathway in pepper.** Enzymes encoded by flavonoid early structural genes (EBGs) are underlined, and those boxed are encoded by flavonoid late structural genes (LBGs).

Flavonoids are secondary plant metabolites that fulfill numerous physiological functions such as pigmentation, health-promoting components, and protection against damage by ultraviolet light and phytopathogens (Lepiniec et al., 2006; Davies et al., 2012). Anthocyanins are soluble flavonoids pigments and its biosynthetic pathway has been elucidated. The enzymes involved in the pathway include phenylalanine ammonia-layse (*PAL*), cinnamate 4-hydroxylase (*C4H*), 4-coumarate: CoA ligase (*4CL*), chalcone synthase (*CHS*), chalcone isomerase (*CHI*), flavanone 3-hydroxylase (*F3H*), flavonoid 3′,5′-hydroxylase (*F3*′*5*′*H*), dihydroflavonols 4-reductase (*DFR*), anthocyanin synthase (*ANS*), and UDP-glucose: flavonoid 3-glucosyltransferase (*UFGT*) (Aza-González et al., 2013; **Figure 1**). Also, an anthocyanin permease (*ANP*) and a glutathione *S*-transferase (*GST*) have been suggested to participate in sequestration of anthocyanin in the vacuole (Mathews et al., 2003).

A protein complex consisting of a *MYB* transcription factor, a basic helix-loop-helix (*bHLH*) protein and *WD40* protein (MBW complex) has been proposed to regulate the expression of structural genes (Broun, 2005; Hatlestad et al., 2015), among which MYB appears to be the major determinant of anthocyanin accumulation (Hichri et al., 2011). By interacting with the promoters of late structural genes (LBGs), the MBW complex conveys significant modulation on the expression of these genes, while its effect on the early structural genes (EBGs) is marginal (Nesi et al., 2001).

Pepper (*Capsicum annuum* L.) is an important horticultural crop cultivated both as an ornamental or vegetable crop. The purple skin color of pepper is preferred by consumers and has become a quality marker for pepper cultivar breeding. The appearance of purple color is due to the abundance of anthocyanin. The anthocyanin biosynthetic pathway has been investigated extensively in pepper fruits, while little research has been done in pepper leaves. Borovsky et al. (2004) isolated *A* (*CaMYB*) from pepper fruits and it was uniquely expressed in various organs of purple pepper as opposed to green pepper. Only the late genes in the pathway are dependent on the expression of *A* (*CaMYB*) in pepper, similar to the results which have been previously investigated in Petunia (Quattrocchio et al., 1999). Stommel et al. (2009) evaluated the expression of anthocyanin structural (*CHS, DFR*, and *ANS*) and regulatory (*MYC, MYBA*, and *WD*) genes in foliar tissue from pigmented and non-pigmented *C. annuum* genotypes, showing that there was no significant difference in the expression of regulatory genes, but pronounced difference in the transcription of structural genes between anthocyanin-pigmented versus and green-colored leaves. These two studies showed inconsistent results regarding the regulation of structural genes in *Capsicum* and also provided incomplete data on the expression of rest genes in anthocyanin biosynthetic pathway in chili pepper, especially in leaves.

Virus-induced gene silencing (VIGS) is a well-established technology in many species to characterize the function of genes, but it has not been applied in studying the anthocyanin biosynthetic pathway in pepper foliage. In this study, we explored the VIGS application in studying the regulation of anthocyanin biosynthetic pathway in pepper leaves. *CaMYB* was knocked down in purple pepper line Z1 using the tobacco rattle virus (TRV) based gene silencing technique (Wang et al., 2013). We examined the anthocyanin content and expression of anthocyanin regulatory gene (*CaMYB, MYC*, and *WD40*) and 12 structural genes in silenced leaves. Based on these results, we propose that *CaMYB* is a key factor in anthocyanin biosynthesis pathway in pepper leaves. In addition, *CaMYB* may be involved in the defense response to pathogens in the purple pepper line.

## Materials and Methods

### Plant Materials and Growth Conditions

The purple pepper line Z1 and green pepper line A3 were provided by College of Horticulture, Northwest A&F University. Z1 is an anthocyanin pigmented cultivar having purple stem and immature fruit under regular growth conditions. Its leaf is purple under the sixth position, and interspersed with green portions above the sixth position. Pepper seeds were germinated on damp sponge at 28°C in the dark for 2 days. Sprouted seedlings were sown into plastic trays containing steam-sterilized growing media in the illumination incubator at day/night temperatures of 23/18°C with relative humidity of 60% and a 16-h light and 8-h dark photoperiod.

### VIGS of *CaMYB* in Pepper

**FIGURE 2:**
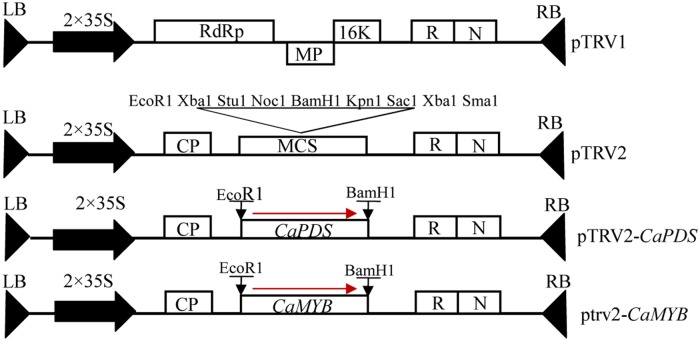
**Schematic representation of the silencing vector.** LB, left borders of the T-DNA; RB, right borders of the T-DNA; 2 × 35 S, two copies of the cauliflower mosaic virus 35 S promoter; CP, coat protein; RdRp, RNA-dependent RNA polymerase; MP, movement protein; 16K, 16 KDa protein; R, ribozyme; N, nos-terminator; MCS, multiple cloning sites.

The pTRV vector and *Agrobacterium tumefaciens* strain GV3101 were prepared for VIGS (Wang et al., 2013; Ji et al., 2014). We cloned *CaMYB* gene in purple pepper Z1 and found that the sequence in the coding region of *CaMYB* is the same as that deposited in the GenBank (AJ608992). Multiple sequence alignment was performed using MEGA6 to reveal the homology among genes involved in anthocyanin biosynthesis from different species. Online software siRNA-scan^1^ was used to avoid off-target silencing (Xu et al., 2006). A 332-bp fragment harboring a conserved and a non-conserved region of *CaMYB* was cloned into the pTRV2 vector by One Step Cloning Kit to yield the pTRV2: *CaMYB* construct. Empty vector (pTRV: 00) was served as a negative control (NC). The pTRV2: *PDS*, which contains the phytoene desaturase sequence to induce a photo-bleaching phenotype, was used as a positive control (**Figure 2**). The primers were shown in **Table 1**. A 10-mL culture of each strain was incubated for 24–36 h at 28°C in LB broth containing 50 mL^-1^ kanamycin, 50 mL^-1^ Gentamicin, and 50 mL^-1^ rifampicin. The primary culture was resuspended into Induction Medium containing 50 mL^-1^ kanamycin, 20 mg mL^-1^rifampicin, 50 mL^-1^ Gentamicin, 200 μM acetosyringone, and was shacked at 28°C for 20–24 h according to Velásquez et al. (2009). The cells were precipitated by centrifugation for 10 min at 3,000 × *g* and resuspended in the same volume of *Agrobacterium* infiltration buffer containing 10 mM MgCl_2_ and 10 mM MES at pH 5.7 till the OD600 reaching 0.5. *A. tumefaciens* GV3101 containing pTRV1 was mixed with GV3101 containing either pTRV2:00 or pTRV2: *CaMYB* in a 1:1 ratio. After mixing them, 400 μM acetosyringone was added.

**Table 1:** List of primers used in this study.

Abbreviation	Forward Primer (5′ to 3′)	Reverse Primer (5′ to 3′)
VIGS-*CaMYB*	GTAAGGTTACCGAATTCGATTGAGGAAGATGTGGCTGC	AGCTCGGTACCGGATCCTTCTTACATTGAAGATGCGTGGA
RT*-CaMYB*	AGATTGCCGGGAAGAACAGCAAAC	TTGCACTTGATGAGAAGGTCCGAG
*PAL*	ATTGATTTTTGCAAGAAATCAATTC	GCTCCACTTTAGCCCCAC
*C4H*	GATTCCTTCCATTCGGTGTT	CCTTTCTCCGTGGTGTCG
*4CL*	CTGGACCAGTGCTGGCAAT	GGTTACGGGGCAAAGAACAA
*MYC*	CAATGGAGCTATAAAGACTAGGAA	GGAAAAGAGAAAGAAACACACATG
*WD40*	GTGTGAATGCTATTGCTTGG	GGAGGAGGACCACTGAAG
*CHS*	GTGGAACCGTTATCCGACTAGCAA	GTATCACTTGGGCCACGGAAAGTA
*CHI*	CCTCCTGGTTCTACACCACC	CTTTGCGGCAGGTGAAACTC
*F3H*	GGCATGTGTGGATATGGACC	CCTCCGGTGCTGGATTCTG
*F3′5′H*	GATGGGGTGGCCGGTGATTG	GCCACCACAACGCGCTCG
*DFR*	AATCGCTCCAGCTGGTCTCATCAT	CTAACACAGGGAAGAGGCTGGTTT
*ANS*	CAAATGCCCACAACCAGAACTAGC	CGCACTTTGCAGTTACCCACTTTC
*UFGT*	GGATGGTGTCAAACAAGGC	GTTCAGTACAACACCATCTGC
*ANP*	GCCAATATGGTGTAAATTCCC	GAGCACATCCACTTTGCTGTG
*GST*	CGGATCCCTCGAGCAGAAAAAACC	CGGATCCCCTTTGTTCATATATG
*UBI*	TGTCCATCTGCTCTCTGTTG	CACCCCAAGCACAATAAGAC

*The restriction enzyme sites added at 5′ end of primers are underlined.*

The plants with the fourth leaf fully expanded were used for VIGS. We punched holes with a needle on both sides of main veins. Then the leaves were infiltrated with a 1 ml syringe without needle. The inoculated plants were grown at 18°C for 48 h in 60% relative humidity under dark and then placed in a growth room at 25°C with a 16-h light/8-h dark photoperiod.

### Pathogen Preparation

Preparation and identification of *Phytophthora capsici* inocula was conducted according to Zhang et al. (2013b). The strain PC of *P. capsici* was grown on potato dextrose agar (PDA) medium in darkness at 28°C. Twenty-one days after the induction of TRV-mediated gene silencing, detaching leaves from non-infiltrated, pTRV2: 00 and pTRV2: *CaMYB* plants were exposed to an 8 mm diameter mycelium plug from *P. capsici*. Then the leaves were placed in a petri dish containing moist filter paper followed by incubation in the dark at 28°C for 3 days.

### RNA Analysis

The total RNA was extracted from the leaves of the inoculated plants of Z1 at the same position as the bleaching leaves of pTRV2: *PDS* plants using the RNA simple kits (Tiangen Biotech, Beijing, China). RNA samples (1 μg) were reverse transcript into complementary DNA (cDNA) using the High Fidelity PrimeScript RT-PCR kit (TaKaRa) according to the manufacturer protocol. The primers of *CaMYB* designed from the specific sequences outside of the silencing region were used for detecting the gene silencing efficiency. Real-time PCR was used to compare the expression of anthocyanin biosynthetic genes including *CaMYB, MYC, WD40, PAL, C4H, 4CL, CHS, CHI, F3H, F3′5′H, DFR, ANS, UFGT, ANP, GST, UBI* between anthocyanin-pigmented and non-pigmented leaves. Sequences related to these genes were identified by comparison with sequences in the GenBank database and the pepper whole-genome sequences database^2^ (Kim et al., 2014). Some of the pepper specific primer sets were adopted from Lightbourn et al. (2007) and Aza-González et al. (2013; Primer sequences were listed in **Table 1**). The relative fold difference in mRNA levels was calculated using the 2^-ΔΔCt^ formula with *CaUbi* as the internal control.

### Measurement of Anthocyanin Production

For extraction of anthocyanin, 5 g leaves of Z1 line were agitated gently in the dark for 24 h at 4°C in 2 mL of 3 M mixture of HCl : water : MeOH, 1:3:16 (v/v/v; Gould et al., 2000). The extracts were centrifuged. High-performance liquid chromatography (HPLC) analysis of anthocyanin was carried out with a HP1200 Liquid Chromatograph equipped with a diode array detector (Agilent Technology, Palo Alto, CA, USA) using the method as described by Stommel et al. (2009). The anthocyanin was identified by comparing both the retention time and the absorption spectrum with those of a commercial standard (delphinidin-3-*p*-coumaroyl-rutinoside-5-glucoside).

## Results

### Phylogenetic Analyses

**FIGURE 3:**
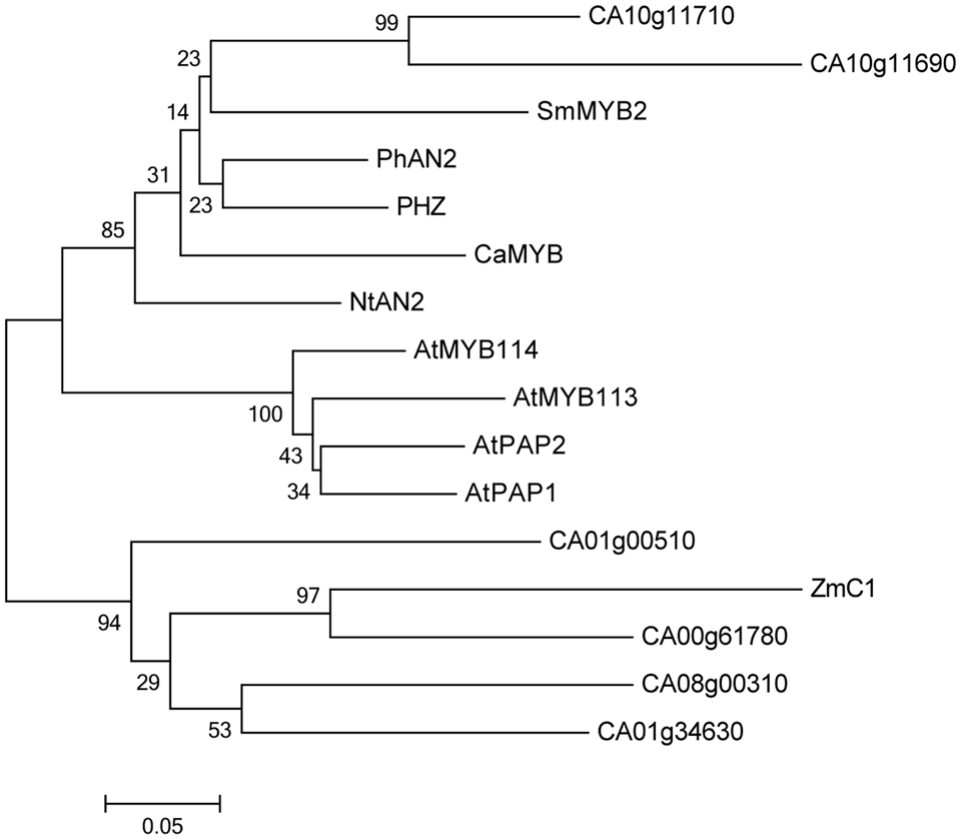
**Phylogenetic analysis of *CaMYB* and other *MYBs*.** Protein sequences used for alignment are as follow: *SmMYB2*, KF727477; *PhAN2*, EF423868.1; *PHZ*, HQ116170; *NtAn2*, FJ472647; *AtMYB113*, NM_105308; *AtMYB114*, NM_105309; *AtPAP1*, NM_104541; *AtPAP2*, NM_105310; *ZmC1*, P10290.1. *Ca, Capsicum annuum* L; *Sm, Solanum melongena*; *Ph, Petunia hybrid*; *Nt, Nicotiana tabacum; At, Arabidopsis thaliana*; *Zm, Zea mays*.

*CaMYB* is clustered with the R2R3 *MYB* transcription factors that are involved in the regulation of anthocyanin biosynthesis in other plant species, such as *Arabidopsis*, maize, petunia, tobacco, and eggplant (**Figure 3**). *CaMYB* is closely related to the subgroup 6 *MYBs* in *Arabidopsis* (Dubos et al., 2010), and the nearest *MYB* is *SmMYB2* in eggplant which belongs to the *Solanaceae*. In pepper genome, there are six genes showing high similarity to *CaMYB*, and the similarity is heterogeneous ranging from 49 to 67% at the protein level.

### Expression of Biosynthetic Genes in both Anthocyanin-Pigmented and Non-Pigmented Pepper

**FIGURE 4:**
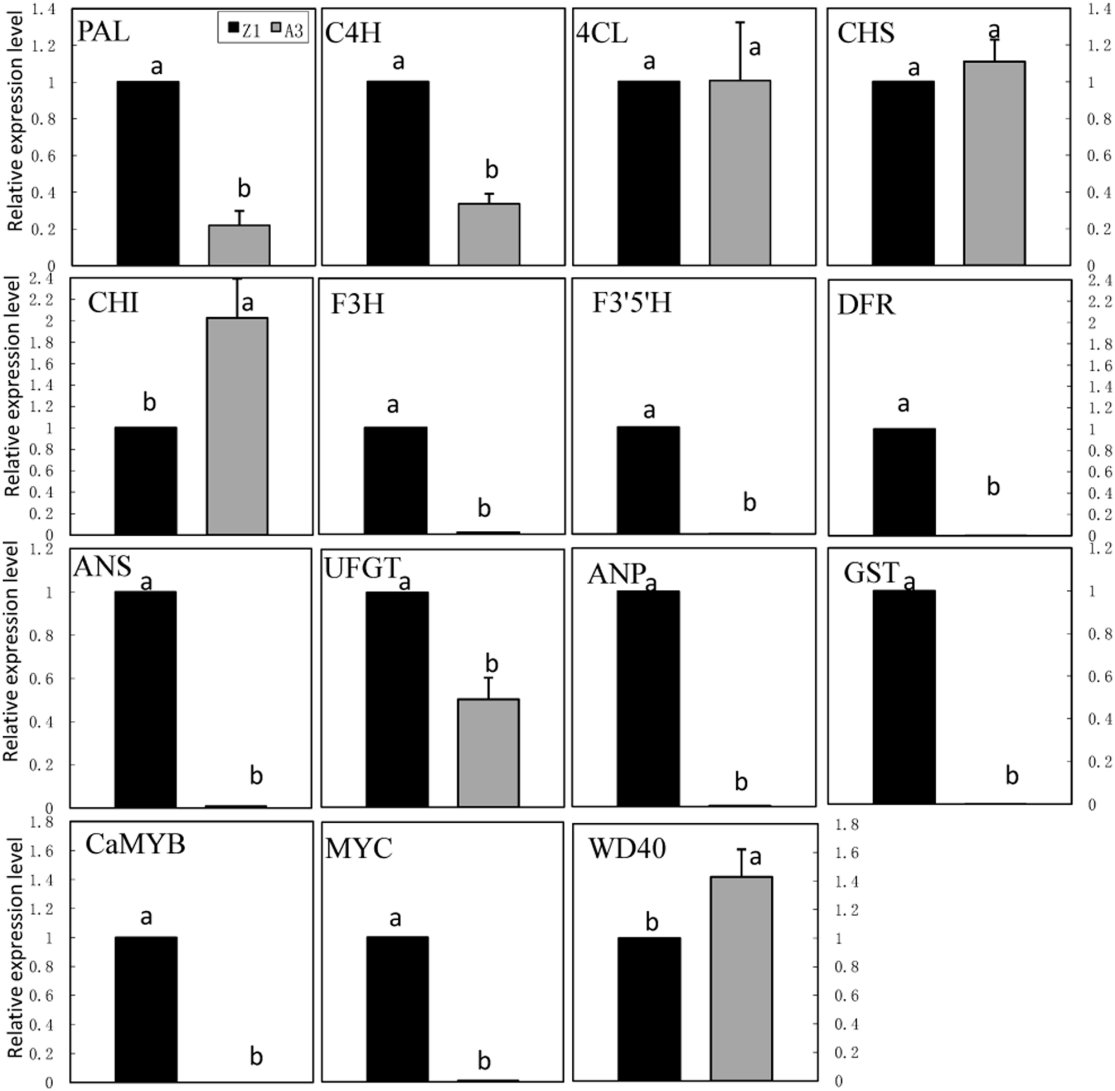
**Anthocyanin biosynthesis and regulatory genes expression in foliage of line A3 and Z1.** Error bars represent the mean ± SD of three independent biological replicates. Bars with different lower case letters in each group indicate significant differences using Duncan’s multiple range test at *p* < 0.05.

The expression of genes involved in the anthocyanin biosynthetic pathway was monitored in leaves of pepper line A3 (non-anthocyanin pigmented) and Z1 (anthocyanin pigmented; **Figure 4**). These genes included phenylpropanold pathway genes (*PAL, C4H, 4CL*), flavonoid pathway genes (*CHS, CHI, F3H, F3′5′H, DFR, ANS, UFGT, ANP*, and *GST*) and regulatory genes (*CaMYB, MYC*, and *WD40*). The transcription level of *CaMYB* was undetectable in A3, but maintained a high level in Z1. The expression of *MYC* was extremely low in A3 as opposed to Z1. On the contrary, the expression of *WD40* was lower in Z1 than in A3. Structural genes could be classified into three groups on the basis of expression pattern. Nine genes including *PAL, C4H, F3H, F3′5′H, DFR, ANS, UFGT, ANP*, and *GST* showed higher expression in Z1 than in A3, whereas *CHI* showed higher expression in A3 than in Z1. The transcript level of *4CL* and *CHS* was similar between A3 and Z1.

### Silencing of *CaMYB* Reduced the Accumulation of Anthocyanins

**FIGURE 5:**
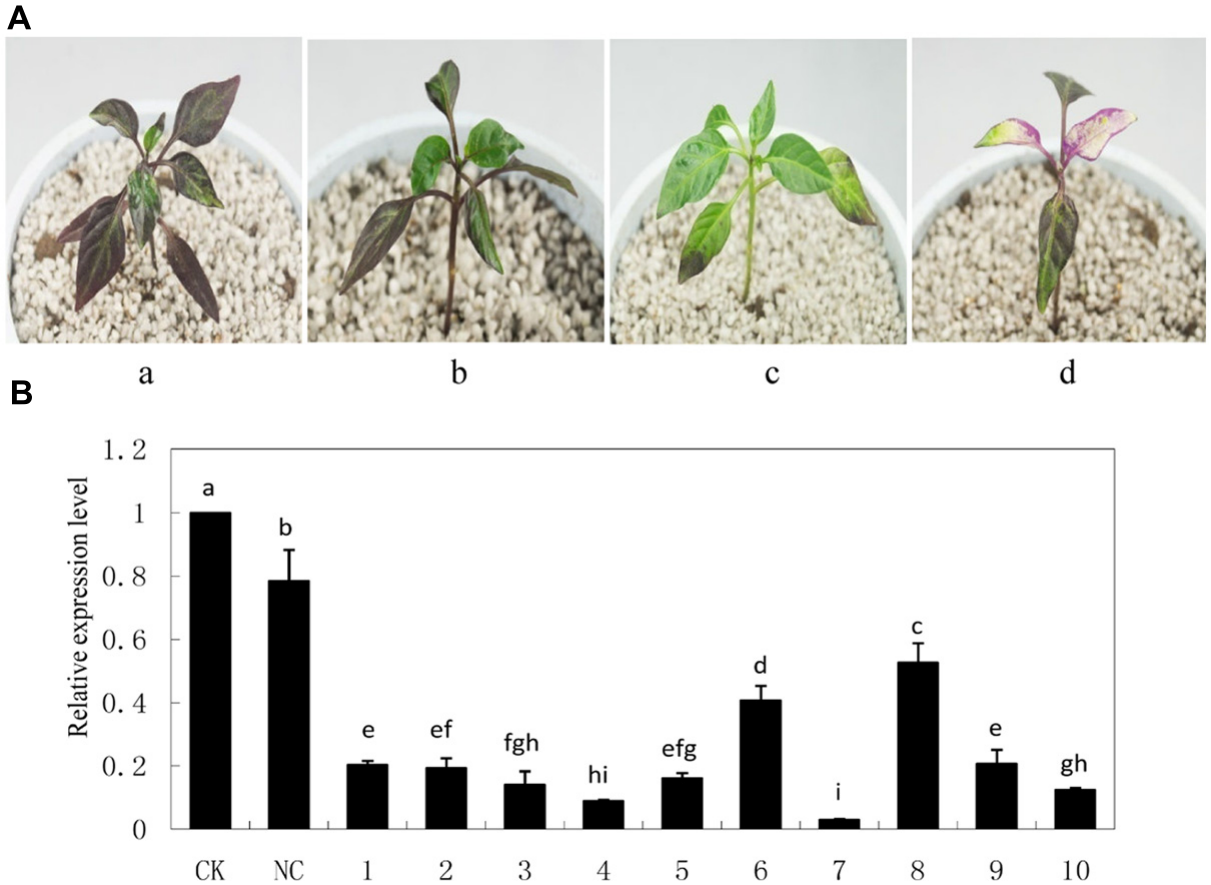
**Silencing efficiency of *CaMYB* in pepper plants using a tobacco rattle virus (TRV)-based Virus-induced gene silencing (VIGS) system. (A)** Phenotypes of pepper plants 21 days after infiltration of different vectors. a: non-infiltrated control (CK); b: negative control (NC) plants (PTRV2:00); c: *CaMYB*-silenced plant (PTRV2:*CaMYB*); d: *PDS*-silenced plant (PTRV2:*PDS*). **(B)** Quantitative real time-PCR analysis of *CaMYB* expression levels in leaves of CK, NC plants and *CaMYB*-silenced plants. Ten silencing plants were analyzed (numbered 1–10). Error bars represent mean ± SD for three technical replicates for each plant. Bars with different lower case letters in each group indicate significant differences using Duncan’s multiple range test at *p* < 0.05.

As an indication of successful silencing, *CaPDS*-silenced plants showed photo bleaching (**Figure 5A**). Z1 leaves infiltrated with pTRV2: 00 (NC) appeared to be less purple colored compared with non-infiltrated Z1 leaves (CK). Z1 leaves infiltrated with pTRV2: *CaMYB* exhibited green coloration, and *CaMYB* abundance was significantly decreased in *CaMYB*-silenced plants compared to the NC (**Figure 5B**).

**FIGURE 6:**
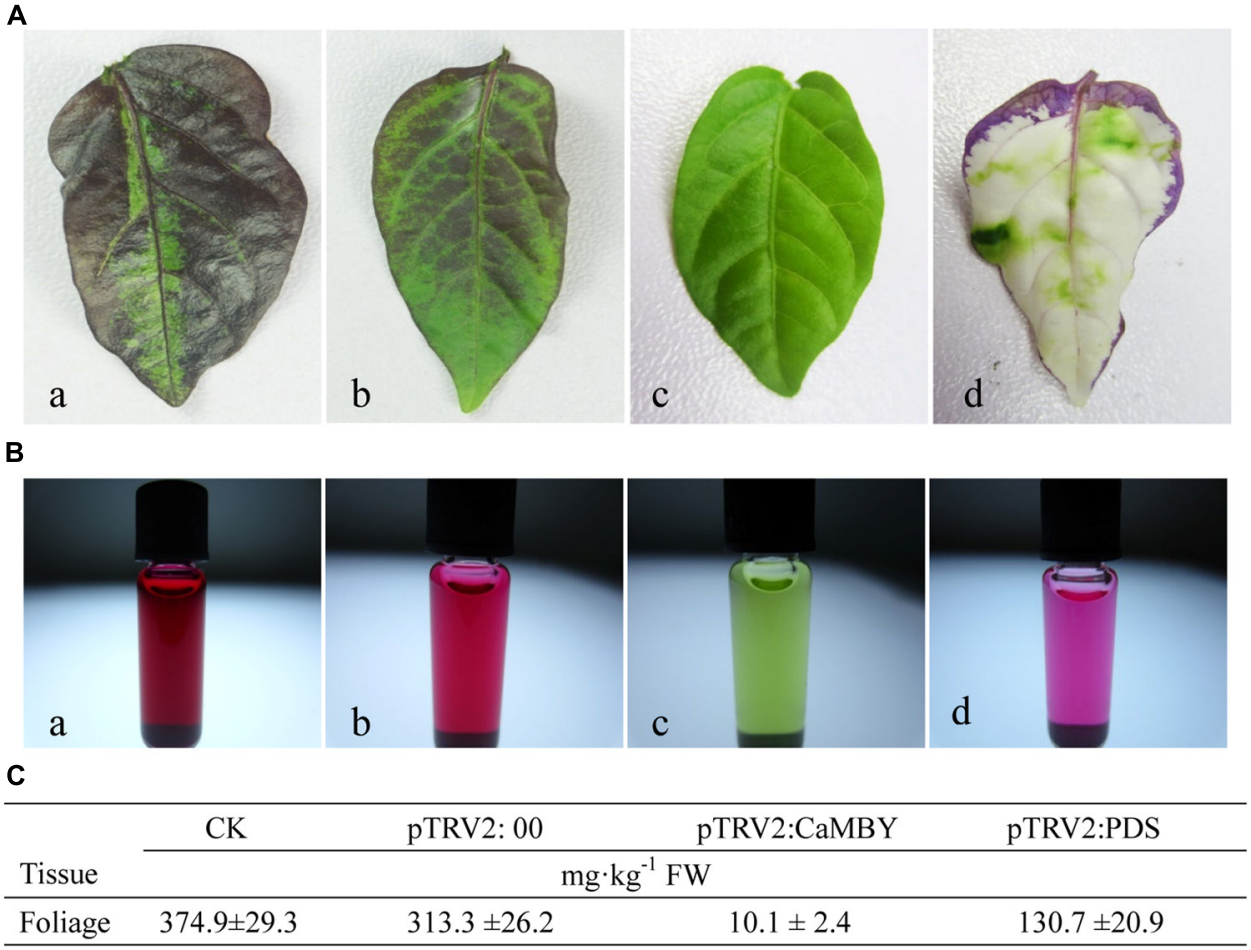
**Variation of anthocyanin accumulation in leaves infiltrated with different vectors. (A)** Leaves were detached from: a: non-infiltrated control (CK); b: NC plants (PTRV2:00); c: *CaMYB*-silenced plant (PTRV2: *CaMYB*); d: *PDS*-silenced plant (PTRV2: *PDS*). **(B)** Extracted solutions from the corresponding four samplings. **(C)** Anthocyanin content of the four samplings by High-performance liquid chromatography (HPLC). Means ± SD (*n* = 3).

Anthocyanin from leaves of CK, NC plants, *CaMYB*-silenced plants and *CaPDS*-silenced plant were extracted and measured by HPLC (**Figure 6**). The color intensity of the extracted solutions was different among four samplings (**Figure 6B**). *CaMYB*-silenced foliage with a green phenotype accumulated far less anthocyanin; a 31- and 37-fold increase of anthocyanin accumulation was detected in NC and in CK, respectively, compared to that in *CaMYB*-silenced leaves (**Figure 6C**).

### Expression of Anthocyanin Biosynthetic Genes in *CaMYB*-Silenced Plants

**FIGURE 7:**
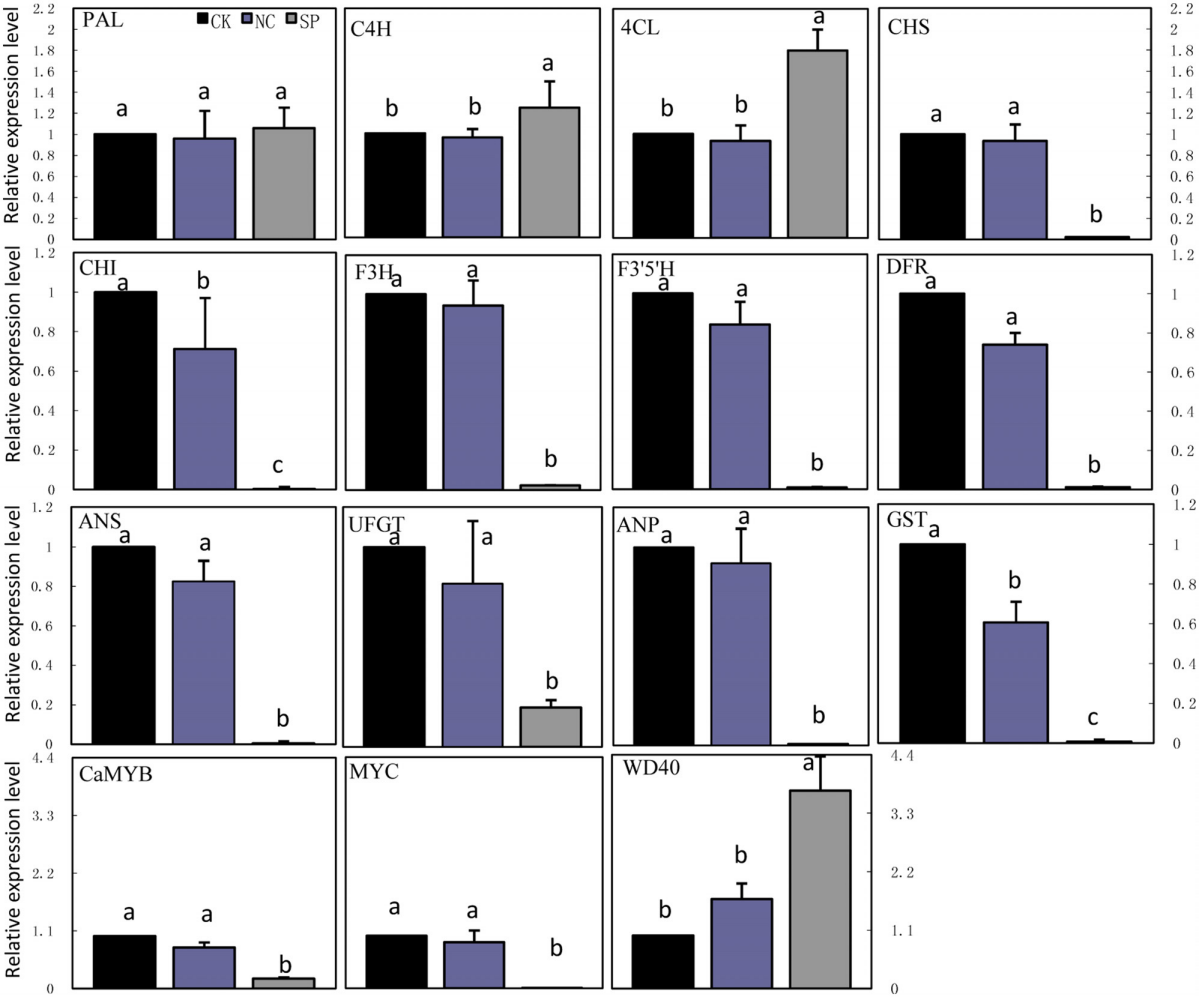
**Expression of anthocyanin biosynthetic genes in foliage infiltrated with different vectors.** CK, non-infiltrated control leaf; NC, negative control leaf (PTRV2:00); SP, *CaMYB*-silenced plants. Error bars represent the mean ± SD of three independent biological replicates. Bars with different lower case letters in each group indicate significant differences using Duncan’s multiple range test at *p* < 0.05.

Real-time quantitative PCR was carried out to examine the transcription of anthocyanin biosynthetic genes in leaves of CK, NC and *CaMYB*-silenced plants (**Figure 7**). The expression of *MYC* was significantly lower in *CaMYB*-silenced leaves than in CK and NC. In contrast, *WD40* showed higher expression in *CaMYB*-silenced leaves than in CK and NC. The transcription of nine structural genes including *CHS, CHI, F3H, F3′5′H, DFR, ANS, UFGT, ANP*, and *GST* were repressed in *CaMYB*-silenced foliage, while *PAL, C4H*, and *4CL* maintained stable and even higher expression in silenced foliage.

### Detached Leaves Inoculation Assays

**FIGURE 8:**
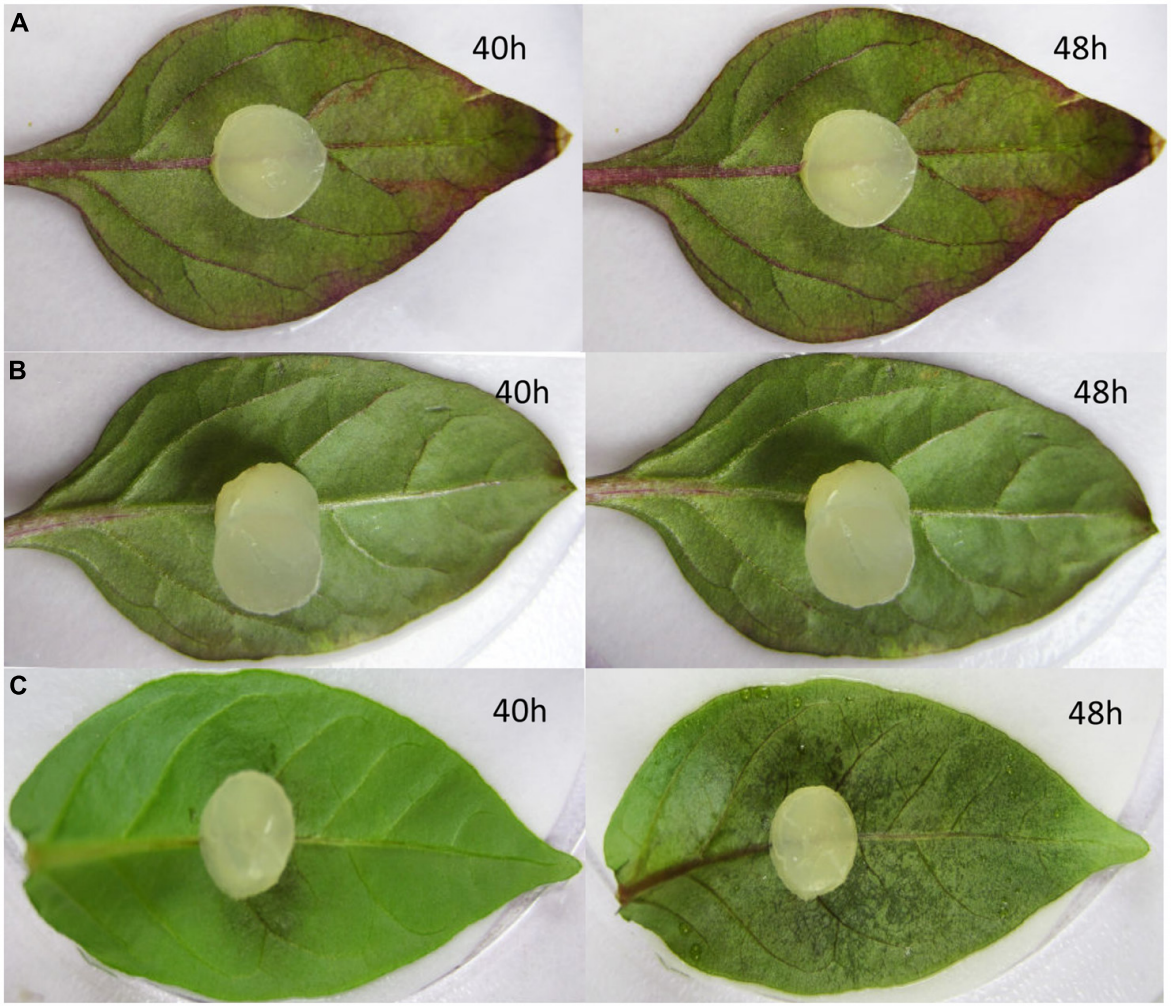
**Disease symptoms developed on the non-infiltrated (CK), empty vector control (pTRV2: 00) and silenced (TRV2: *CaMYB*) leaves infected by *Phytophthora capsici*. (A)** Non-infiltrated control; **(B)** leaves were infiltrated with TRV empty vector; **(C)** leaves were infiltrated with TRV2: *CaMYB* vector. Pictures were taken at 40 and 48 h after the inoculation.

To determine the role of *CaMYB* in the basal defense, detached leaves from CK, NC, and pTRV2: *CaMYB* plants were exposed to an 8 mm diameter mycelium plug from *P. capsici* 21 days after the performance of the VIGS. Water-soaked lesions occurred 40 h after inoculation on the silenced Z1 leaves, and 8 h later the whole leaves became water-soaked (**Figure 8**). In contrast, water-soaked lesions were visible on CK and the NC plants at 72 h after inoculation and the symptoms developed slowly.

## Discussion

It has been reported that purple pepper leaves can enhance the photosynthesis and alleviate the oxidative stress. The anthocyanin epidermal layer may limit photo-inhibitory effects on PSII, the formation of reactive oxygen species and oxidative cellular damages of *Tradescantia pallida* leaves, supporting the role of anthocyanin pigments in the regulation of photosynthesis for excess absorbed light irradiance (Dewez and Perreault, 2013). Also, pepper seedlings can be consumed fresh and the purple pigments adds nutritive value. Thus it is worthwhile to expand the understanding of anthocyanin biosynthetic pathway in leaves.

Phylogenetic analysis indicates that *CaMYB* may execute similar functions as that in other plant species (**Figure 2**). In *Arabidopsis*, plants harboring a silencing construct targeting *MYBs* showed decreased *MYB* gene expression and visible depletion of pigmentation (Gonzalez et al., 2008). In this study, silencing of *CaMYB* resulted in considerable loss of anthocyanin accumulation in pepper leaves (**Figures 5** and **6**). Likewise, disabling of *CaMYB* in pepper fruits reduced the anthocyanin content with lower efficiency (Aguilar-Barragán and Ochoa-Alejo, 2014) in comparison with the results from leaves in this study. These evidence supports that *MYBs* are essential for anthocyanin production in various organs, including pepper leaves.

The transcription levels of *CaMYB* and *MYC* were undetectable in non-pigmented foliage (A3), but an increase in expression of *WD40* was observed in A3 compared with Z1 (**Figure 4**), which is inconsistent with the results from Stommel et al. (2009) probably owing to different lines used in the two studies. The expression of *MYC* was significantly lower in *CaMYB*-silenced leaves, whereas *WD40* showed the opposite pattern (**Figure 7**). It has been noted that R2R3-*MYB* gene alone is capable of regulating the expression of the *bHLH* (*MYC*) partner (Baudry et al., 2006; Albert et al., 2014). *MYB* may induce the expression of *MYC*, co-regulating the accumulation of anthocyanin. *WD40* transcription factor serves as a scaffold that allows the interactions between different *MYC* and *MYB* proteins, such as Transparent Testa Glabra1 (*TTG1*) in *Arabidopsis thaliana* (Gonzalez et al., 2008). In *Petunia* sp., *AN11* (*WD40*) protein activated *AN2* and *AN4* (*MYB* domain proteins; Quattrocchio et al., 1993; Spelt et al., 2000). More direct evidence is needed to clarify the mechanisms of MBW complex in the anthocyanin biosynthesis in pepper foliage.

Based on the different expression mode of *A* (*CaMYB*)and four anthocyanin biosynthetic related genes in both anthocyanin-pigmented and non-pigmented pepper lines, Borovsky et al. (2004) concluded that the early genes (*CHS, CHI*) in the flavonoid pathway are regulated independently of *A*, while expression of the late genes (*DFR, ANS*) is *A*-dependent. In this study, the expression modes of the anthocyanin biosynthesis related genes in Z1 and A3 indicated that the transcription of LBGs and EBGs except *CHS* and *CHI* may be dependent of *CaMYB* (**Figures 1** and **4**). This observation was further confirmed by VIGS showing that the expression of flavonoid pathway genes were altered in *CaMYB*-silenced plants (**Figure 7**). The expressions of *CHS, CHI, F3′5′H, DFR*, and *3GT* (*UFGT*) genes were decreased in pepper fruits transformed with the TRV2- *MYB* construct (Aguilar-Barragán and Ochoa-Alejo, 2014), which also indicated that *CHS, CHI* were dependent on the expression of *MYB* (*CaMYB*). The transcription of *F3H* did not differ between fruits treated with either the empty TRV2 or TRV2- *MYB* and the normal fruits, indicating that *F3H* might be modulated in a different manner in pepper leaf from that in pepper fruits.

It was noted that the flavonoid pathway genes were effectively suppressed, while phenylproanold pathway genes showed a stable (*PAL*) or an even higher (*C4H, 4CL*) expression level in *CaMYB*-silenced leaf (**Figure 7**). Presumably *CaMYB* directly activates transcription of *CHS*, which is the first gene with lower expression in the anthocyanin biosynthetic pathway in *CaMYB-*silenced plants. Espley et al. (2007) identified the interaction signature motif in *MdMYB10*, showing that the protein interacts with *bHLH* proteins to activate transcription of *DFR* in apple (Espley et al., 2007). In *A. thaliana*, ternary complex (*MYB*–*MYC*–*WD40*) is important in stimulating anthocyanin formation by activating transcription of *DFR* and *ANS* along with trichome formation (Nemie-Feyissa et al., 2014). Here, VIGS provides a useful tool to preliminary screen the target sites of transcription factors, however, sophisticated experiments are needed to identify the authentic targets of transcription factors.

*CaMYB*-silencing plants rendered susceptibility to *P. capsici* (**Figure 8**). It has been observed that anthocyanins often served as the first line of defense against pathogen attacks (Wang et al., 2011). Disruption of *Del*/*Ros1* partially reduced the accumulation of anthocyanins on purple tomato fruits, and areas with less anthocyanin were more susceptible to *Botrytis cinerea* than purple areas (Zhang et al., 2013a). In potato, common scab resistance was correlated with the expression of *MYB* and three *bHLH* genes, indicating that they might be involved in the regulation of the defense response against the common scab pathogen (Tai et al., 2013). Presumably *CaMYB* gene plays a role, at least partially, in resistance against *P. capsici*. Therefore effective deployment of *CaMYB* may aid to develop pepper cultivars with favorable anthocyanin production and resistance level.

## Conclusion

This study provides a more comprehensive study on the regulation of anthocyanin biosynthetic genes in pepper leaves. Differential gene expression between leaf of A3 and Z1 and in pepper leaves by VIGS suggests that *CaMYB* in *C. annuum*. L regulates the expression of all the flavonoid pathway genes. The gene expression analysis in *CaMYB*-silenced leaves demonstrates that the transcription factor of *CaMYB* plays a vital role in the MBW activation complex for anthocyanin accumulation, and MYC might be positively regulated by *CaMYB*, while *WD40* might be negatively regulated by *CaMYB* in pepper leaves. Furthermore, detached leaves inoculation assay from VIGS system revealed that *CaMYB* may play an important role in resistance to *Phytophthora capsici Leonian* in purple pepper. The evidence presented in this study in conjunction with results acquired from further research will help clarify the mechanisms of MBW complex in the anthocyanin biosynthesis and defense response in pepper and other plants.

## Author Contributions

ZZ, D-WL, and Z-HG conceived the research. ZZ, D-WL, J-HJ, H-XZ performed the research. ZZ, D-WL, YL performed interpretation of data. ZZ, D-WL, and H-XZ took the photos. ZZ, D-WL and Y-XY performed statistical analyses. ZZ, D-WL wrote the paper. ZZ, D-WL, and Z-HG revised the paper. D-WL and Z-HG provided the materials and resources for the research. ZZ, D-WL, W-GC, and Z-HG performed the integrity of the work. All authors read and approved the final manuscript.

## Conflict of Interest Statement

The authors declare that the research was conducted in the absence of any commercial or financial relationships that could be construed as a potential conflict of interest.

## Footnotes

1 http://bioinfo2.noble.org/RNAiScan/RNAiScan.htm

2 http://peppergenome.snu.ac.kr
